# Accelerometer-based wireless body area network to estimate intensity of therapy in post-acute rehabilitation

**DOI:** 10.1186/1743-0003-5-20

**Published:** 2008-09-02

**Authors:** Stéphane Choquette, Mathieu Hamel, Patrick Boissy

**Affiliations:** 1 Research Centre on Aging, Health and Social Services Centre, Sherbrooke Geriatric University Institute, Quebec, Canada; 2 Faculty of Physical Education and Sports, Department of Kinesiology, Université de Sherbrooke, Sherbrooke, Quebec, Canada; 3 Center of Excellence in Information Engineering, Université de Sherbrooke, Sherbrooke, Quebec, Canada

## Abstract

**Background:**

It has been suggested that there is a dose-response relationship between the amount of therapy and functional recovery in post-acute rehabilitation care. To this day, only the total time of therapy has been investigated as a potential determinant of this dose-response relationship because of methodological and measurement challenges. The primary objective of this study was to compare time and motion measures during real life physical therapy with estimates of active time (i.e. the time during which a patient is active physically) obtained with a wireless body area network (WBAN) of 3D accelerometer modules positioned at the hip, wrist and ankle. The secondary objective was to assess the differences in estimates of active time when using a single accelerometer module positioned at the hip.

**Methods:**

Five patients (77.4 ± 5.2 y) with 4 different admission diagnoses (stroke, lower limb fracture, amputation and immobilization syndrome) were recruited in a post-acute rehabilitation center and observed during their physical therapy sessions throughout their stay. Active time was recorded by a trained observer using a continuous time and motion analysis program running on a Tablet-PC. Two WBAN configurations were used: 1) three accelerometer modules located at the hip, wrist and ankle (M3) and 2) one accelerometer located at the hip (M1). Acceleration signals from the WBANs were synchronized with the observations. Estimates of active time were computed based on the temporal density of the acceleration signals.

**Results:**

A total of 62 physical therapy sessions were observed. Strong associations were found between WBANs estimates of active time and time and motion measures of active time. For the combined sessions, the intraclass correlation coefficient (ICC) was 0.93 (P ≤ 0.001) for M3 and 0.79 (P ≤ 0.001) for M1. The mean percentage of differences between observation measures and estimates from the WBAN of active time was -8.7% ± 2.0% using data from M3 and -16.4% ± 10.4% using data from M1.

**Conclusion:**

WBANs estimates of active time compare favorably with results from observation-based time and motion measures. While the investigation on the association between active time and outcomes of rehabilitation needs to be studied in a larger scale study, the use of an accelerometer-based WBAN to measure active time is a promising approach that offers a better overall precision than methods relying on work sampling. Depending on the accuracy needed, the use of a single accelerometer module positioned on the hip may still be an interesting alternative to using multiple modules.

## Background

Post-acute rehabilitation is a key component of the health care delivery system, yet we know little about the active ingredients of the rehabilitation process that produce the best outcomes [1]. Rehabilitation care has been compared to a black box [2] or a Russian doll [3]. The measurement of rehabilitation interventions is thus acknowledged to be amongst the major methodological challenges to conducting research in this area [1].

Evidence suggests that the amount of therapy during rehabilitation shares a dose-response relationship with functional outcomes. In fact, a meta-analysis has reported increases in functional recovery of stroke patients with increased hours of therapy throughout the length of stay [4]. In addition, more hours of therapy each day may shorten the length of stay of orthopedic and stroke patients [5].

Regarded as the most active component of rehabilitation, total time of therapy has been referred to as the "intensity" of rehabilitation [4,6,7]. This denomination may be misleading [8] since time spent in organized therapy is probably not an accurate portrait of the therapies intensity and contents and their link with functional outcome changes. It has been suggested that investigations on determinants of post-acute rehabilitation processes should focus on specific aspects of therapy instead of total time of therapy [9]. The assessment of the effectiveness of rehabilitation procedures has been limited to the laboratory setting; relatively little is known about rehabilitation in real-life situations.

Active time, or the time during which a patient is physically active, has been suggested as a key factor in functional recovery [10,11]. Large inter-individual variations in the time in which a patient is physically active are to be expected because of a patient's motivation, health status, physical capabilities and medication [4]. Such variations have been reported in previous studies [12,13] and could mean that active time may be a better indicator of rehabilitation intensity than total time of therapy. Large-scale longitudinal studies are necessary to explore associations between active time and functional recovery.

In the past, specific aspects of therapy have been documented using retrospective analysis of medical records [4,14,15] or observational methods [10-13]. Observational studies are conducted by having a trained observer follow the patient for a predetermined period of time to record the duration of activities and/or mobilization. Observational approaches like work sampling [10,11] and time and motion [12,13] have been used in rehabilitation. Time and motion (TM) is recognized as the most precise approach to collect valid data on clinical practices in the health field [16]. Unfortunately, data collection and processing in time and motion studies are both resource-consuming. Consequently, observational studies in rehabilitation have only been descriptive in nature and conducted for only a few consecutive days [10,11,13,17].

Methods more efficient than observation are needed to measure active time in rehabilitation. Miniature, wireless, and wearable technology offers a tremendous opportunity to address this issue. Recent technological advances in integrated circuits and wireless communications have led to the development of Wireless Body Area Networks (WBANs). Wireless body area networks may be a viable alternative to measure active time. They can include a number of physiological sensors depending on the end-user application, are well suited for ambulatory monitoring and provide specific information about an individual's behavior without using complex laboratory equipment and without interfering with the person's natural behavior [18].

WBANs have been used in at least two studies to monitor heart rate in rehabilitation settings. MacKay-Lyons et al. (2002) observed that only a mean of 2.8 ± 0.9 min and 0.7 ± 0.2 min, for physical and occupational therapy sessions respectively, were spent in a targeted heart rate zone that could illicit an improvement in cardiovascular capacity [19]. Gage et al. (2007) also found that there were little differences in heart rate between the execution of low and high therapeutic activities [13]. Consequently, it was concluded that cardiovascular stress does not reflect therapeutic activities in rehabilitation [13,19].

Kinematics has been suggested as a better alternative to estimate mobilization and active time in rehabilitation [13]. Accelerometers have gained recognition as an interesting way to measure physical activity in the population [20]. They can record intensity and duration of activities through movement accelerations [21]. Therefore, they may constitute a convenient approach to measure active time during therapy sessions.

In order to alleviate the burden of observational methods in the investigation of active time of therapy, the primary objective of this study was to compare, with patients during real life physical therapy, time and motion measures with estimates of active time (i.e. the time during which a patient is active physically) obtained with a wireless body area network (WBAN) of 3D accelerometer modules positioned at the hip, wrist and ankle. The secondary objective was to assess the differences in estimates of active time when using a single accelerometer module positioned at the hip.

## Methods

### Study design

**Figure 1:**
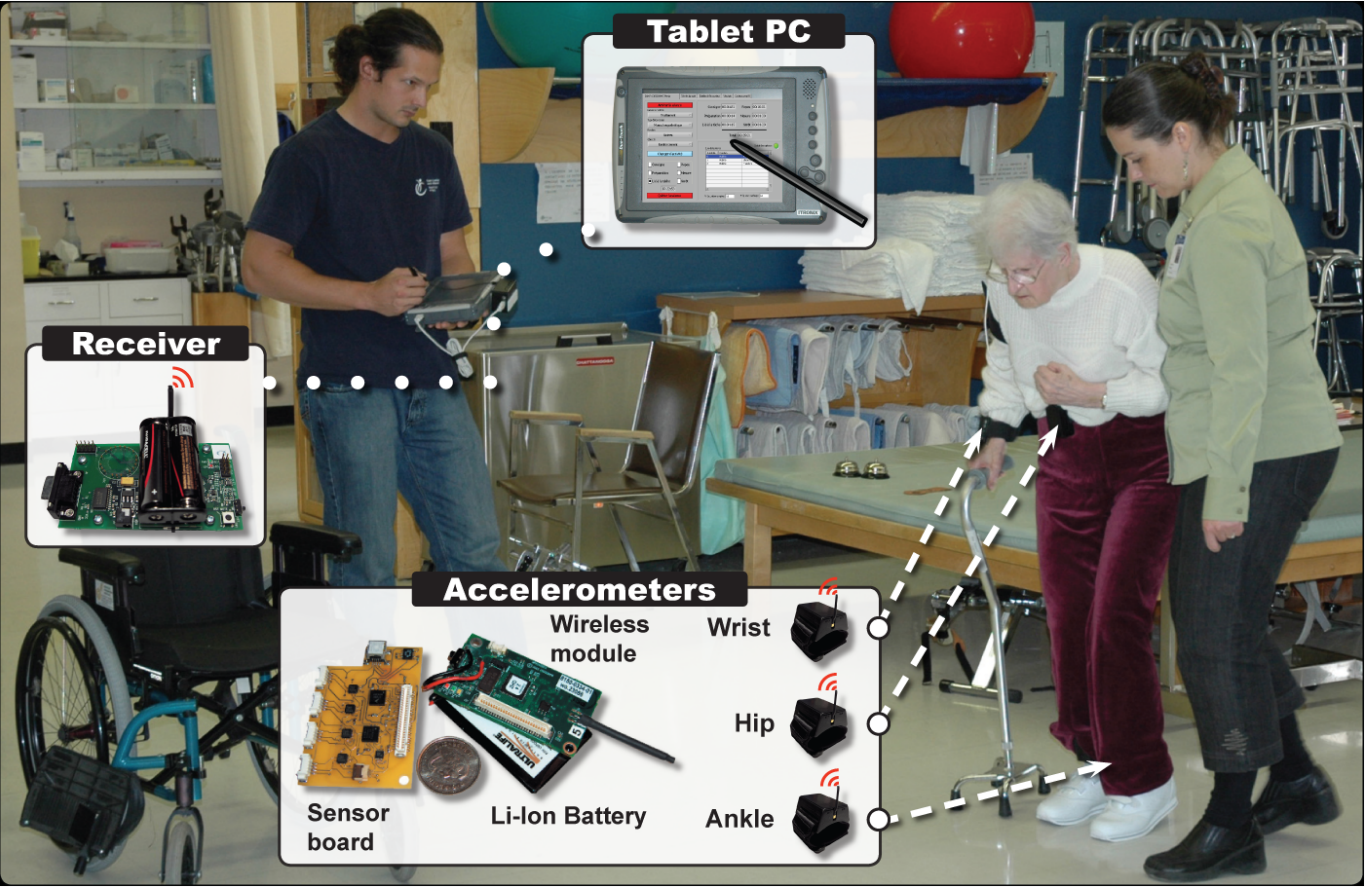
Time and motion observations and recording of body accelerations. The WBAN used in this study was comprised of three 3D accelerometers modules. Signals recorded by accelerometers were transmitted to a receiver located on the Tablet-PC. The Tablet-PC recorded WBAN's data in background, while an observer noted time and motion parameters of the session. All data was synchronized on a common timeline.

Participants were observed continuously during their physical therapy sessions while accelerometer signals from a WBAN were recorded simultaneously (Figure 1). A sample of convenience was recruited from the Intensive Functional Rehabilitation Unit (IFRU) of the Health and Social Services Centre – Sherbrooke Geriatrics University Institute. Patients were eligible to participate if they were over 65 years old and were admitted to the IFRU following discharge from an acute hospital. Patients presenting cognitive deficits that would compromise their capacities to understand the nature of their participation in the study were excluded.

Participants were recruited about one week after their admission to the IFRU. Their participation in the study began immediately after written consent was obtained and continued until discharge, with three to five physical therapy sessions observed each week. All observations were conducted by the same observer. Ten minutes before each physical therapy session, three wireless accelerometers modules were attached to the patient by the observer. Recordings began as soon as the therapist made contact with the patient in the therapy unit. The therapy was conducted by the clinicians without any intervention from the observer.

Participants were evaluated prior to the beginning of the observations using a battery of standardized clinical tests that included variables such as functional autonomy (SMAF) [22], balance (Berg) [23], Timed-up-and-go (TUG) [24], and the 5m-Walk test [25]. The SMAF (Functional Autonomy Measurement System) is designed for clinical use in connection with a home support program or for admission and monitoring of clients in geriatric services and residential facilities. The median total SMAF score varies according to living environment (13.5 own home, 29.0 intermediate resources and 55.0 long-term care institutions) and nursing care time. The institutional review board of the HSSC-UIGS approved this study. Informed consent was obtained for all participants.

### Time and motion measurements

Observations were recorded using a continuous TM analysis program running on a Tablet-PC (Intronix DuoTouch). Each session was divided into groups of activities according to the treatment objectives and methods used. The classification used to divide the therapeutic activities is adapted from the classification proposed by Dejong [26]. It is a simplified version of a grid that has been validated in a previous study [12]. This grid was based on the theoretical construct of the Functional Autonomy Measurement System (SMAF) [27]. It contained a total of 38 categories of activities covering frequent objectives targeted by interventions in physical therapy, occupational therapy and speech-language therapy (e.g. use stairs, dress oneself). In the present study, observations were made only in physical therapy sessions. Therefore, fewer categories of activities were needed.

Based on frequency analyses made from data collected in a previous study in post-acute rehabilitation, we reduced the original grid to 8 categories. Those categories were: Antalgic therapy (application of ice or warmth, massage, ultra-sound, etc.), Balance (staying upright for a given amount of time), Gait (all walking activities performed inside the hospital, on the floor or on a treadmill, using whatever walking aids necessary), Outdoor walking (walking outside of the hospital walls), Reinforcement (activities that aimed to strengthen, sometimes with additional resistance, specific muscle groups, either with repetitive movements or isometric contractions), Prosthesis (all activities related to the installation or the adjustment of a prosthesis), Stairs (climbing stairs, up and down), Weight bearing (various activities where the goal is to put weight on the limbs) and Others (all other activities that does not fit in any of the other 7 categories).

For each activity, the observer classifies the time spent by the patient as *active time *or *passive time*. Active time is defined as the time during which the patient is physically active, in preparation or execution of a task-oriented action. The patient does not have to be in company of the therapist. By implication, the presence of the therapist does not mean systematically that the patient is "active". During passive time, the patient is not physically active or receiving treatment. For example, the patient is "passive" when he sits on a chair, resting between two activities. He is still "passive" when the therapist is explaining to him the objective of an upcoming activity. However, he is considered "active" as soon as he begins to rise from its chair to prepare for an activity. Therefore, a patient is considered "active" if he is walking to reach a flight of stairs, even if the activity is "Stairs". Finally, time clocks for active and passive time were incremented by the observer.

### WBAN and estimates of active time

**Figure 2:**
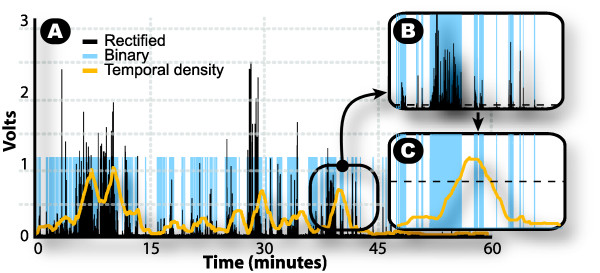
Estimation of active time with accelerometers signals. The three steps of signal transformation are presented in A: 1-Rectified signal, 2-Binary signal and 3-Temporal density. In B, the rectified signal is transformed in a binary signal: all samples above 0.015 Volts (dotted line) are given a value of "1", while samples below equal zero. In C, temporal density is obtained by filtering binary signal with a rolling window of 10 sec. Then, all samples above 0.5 (dotted line) is cumulated to give the active time estimate.

The WBAN used in this study is configured with three wireless sensor modules, each comprised of a custom sensor board with an embedded three axial (3D) accelerometer (LIS3L02AQ, STMicroelectronics) and a communication module with a microcontroller and analog-to-digital converter (MICAz Crossbow Technology). The WBAN system used in this study has been described elsewhere [28]. Data was sampled and recorded at 50 Hz. Wireless sensor modules were embedded in bracelets that could be attached to the body. Modules were installed on the dominant hand, the contra lateral ankle and on the right hip. Active time was estimated by extracting the temporal density of the acceleration signals (Figure 2). Raw signals from separate axes and modules were combined, low-pass filtered (Butterworth, 1 Hz, 2^nd ^Order), rectified and high-pass filtered (Butterworth, 5 Hz, 2^nd ^Order). Data was then saturated in order to obtain a binary signal. Samples with a value above the noise baseline (15 mV), were considered as movements and were associated with a logic high state (ones). All other samples were modified to a low state (zeros). A rectangular rolling window with a length of 10 seconds extracted the envelope of the binary signal and attenuated isolated peaks of acceleration which were not related to physical activity, thus generating a signal with values varying between 0 and 1. Another threshold, optimized with data from first session observed, was fixed at 0.5. Every sample equal or above 0.5 was considered as movement. The cumulative of these samples yielded an estimate of active time.

### Variables and statistical analysis

The variables are 1) the measure of active time, obtained by TM observations and 2) the estimates of active time obtained with WBANs' recording of body acceleration. Two WBAN configurations were used to evaluate the potential of accelerometers to estimate active time in rehabilitation: M3) three accelerometer modules located at the hip, wrist and ankle, and M1) one accelerometer located at the hip.

Descriptive statistics were used to document variability in measurements across subjects. Intraclass correlation coefficients (ICC) were used to evaluate the association between estimates and measurements of active time. The difference of agreement between the reference measure of active time (Time motion) and estimates (M3 and M1) were evaluated with Bland-Altman plots [29,30]. Finally, Paired-Sample T Tests were used to assess the differences in the degree of agreement of the measure of active time between M3 and M1.

Level of agreement between active time measured with both methods (WBAN and TM) was set at 20%. Since there is no actual gold-standards in for the accurate measurement of active time in rehabilitation, setting a critical margin of agreement between methods is somewhat arbitrary. However, a level of agreement of 20% appears to be a reasonable cut level inside which the use of a WBAN, in this particular context, would be justified. This assertion is based on available literature that compares work-sampling methods and TM analysis in the health services literature [16,31,32]. Reported mean error between TM and work sampling is at least 20%, in the most favorable activities. Level of agreement is generally far worse. Therefore, a level of agreement of 20% would assure that our WBAN-based system performs better than what is considered in the present as one of the best available compromise between accuracy and feasibility. This would yield preliminary support to further research efforts in that field.

Statistical analyses were computed using cumulative data from therapy sessions and segmented activities during therapy sessions. Analyses and graphs were completed using SPSS 15.0 program (Chicago, IL). The statistical significance threshold was set at p ≤ 0.05.

## Results

Five patients (77.4 ± 5.2 y) with 4 different admission diagnoses were recruited in this study. The participants' clinical profiles are presented in Table 1. Disability scores on the SMAF scale [22] varied from -19 to -40 (mean -32.4 ± 8.4 on a total of -87) and were linked to physical impairments secondary to stroke, lower limb fracture, amputation and immobilization syndrome. In all the patients, the use of a walker was needed to perform their daily activities. On the Berg balance scale, balance disability varied from 5 to 37 out of a possible total score of 56.

**Table 1:** Clinical characteristics of participants at baseline evaluation and description of observations.

CLINICAL						
	S1	S2	S3	S4	S5	All

Age	72	73	78	79	85	77.4 ± 5.2
Diagnostic	Immob. Syndrome	Fractured femur	Fractured hip	Femoral amput.	Fractured hip Stroke	NA
SMAF (0 to -87)	-35	-30	-38	-19	-40	-32.4 ± 8.4
Berg (0–56)	5	10	29	37	16	19.4 ± 13.3
TUG (sec)	*	56.7	34.6	61.0	82.0	58.6 ± 19.4
5 m walk (sec)	*	22.1	12.5	*	18.3	17.6 ± 4.8

OBSERVATIONS						

N of Sessions	8	12	10	12	20	62
Total time (min)	58.4 ± 9.1	52.0 ± 3.8	57.2 ± 12.1	43.5 ± 6.2	59.5 ± 8.7	47.8 ± 12.2
Active time (min)	39.8 ± 11.3	33.2 ± 6.5	19.3 ± 9.3	33.0 ± 7.6	33.6 ± 9.9	27.0 ± 11.1
Density (%)	67.8 ± 13.3	63.9 ± 12.0	34.1 ± 14.4	75.5 ± 11.5	48.2 ± 8.7	56.8 ± 18.1

Values are presented as mean ± SD. All patients needed to use a walker in order to perform mobility tests, like TUG and the 5-m walk. An asterisk (*) indicates that the patient was unable to accomplish a given test at baseline evaluation. Mean values for performance tests were calculated only on available data. Density represents the proportion of total active time on total time of therapy for all sessions. Immob. Syndrome is for Immobilization Syndrome. Femoral amput. is for Femoral Amputation.

A total of 62 physical therapy sessions were observed (Table 1). The total number of observed sessions for each patient varied from 8 to 20, with a mean of 12 ± 5.2 sessions. Variations in the number of sessions reflect different lengths of stay at the IFRU. Time and motion results showed that the mean active time recorded per session was 27.0 ± 11.1 min for a mean total time of 47.8 ± 12.2 min. Density of therapy, the ratio of active time on total time, was 56.5% for combined sessions. In addition, 295 activities were observed for four patients (the segmentation of sessions was not possible for subject 1 because software malfunction). Only 8 categories of activities had sufficient occurrences (N ≥ 6) to allow analyses. Other activities represented about 4% of the total number of activities (N = 13) and were regrouped under the category "Others".

**Figure 3:**
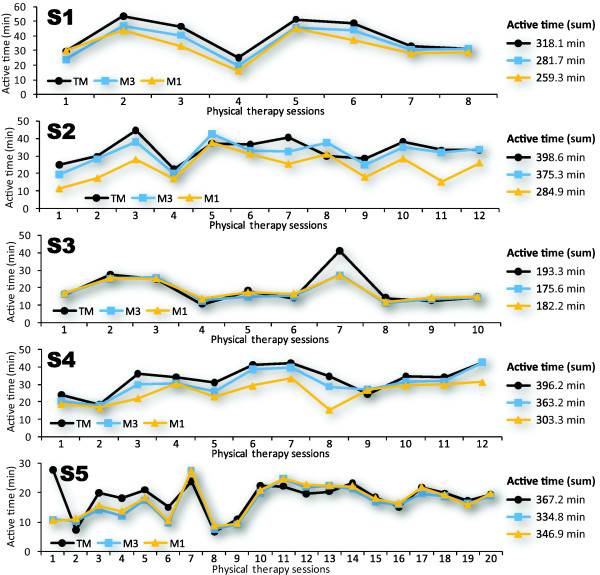
Measure and estimates of active time of therapy sessions throughout the length of stay for each subject.

Figure 3 presents fluctuations in active time during the entire length of stay in the rehabilitation unit, paralleled with estimates of active time from M1 and M3. Cumulative value of active time for each method is presented on the right side of the figure. Estimates systematically underestimate active time, when compared to TM measurements. The mean percentage of differences between measure and estimate is -8.7% ± 2.0% (range: -5.85% to -11.44%) for M3 and -16.4% ± 10.4% (range: -5.53% to -28.52%) for M1.

**Figure 4:**
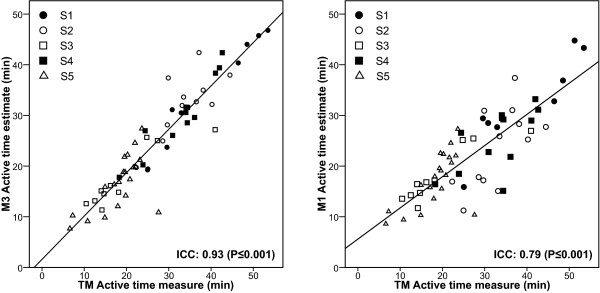
Association between estimates of active time and measure of active time for observed sessions. Intraclass correlation coefficient between accelerometers' estimates and measurement of active time are presented in the lower right corner of each scatter plot. 95% Confidence interval of ICC was 0.89 to 0.96 for M3 and 0.68 to 0.87 for M1.

Scatter plots of estimates by measure of active time are presented for observed sessions in Figure 4. For combined sessions, ICC was 0.93 (P ≤ 0.001) for M3 and 0.79 (P ≤ 0.001) for M1. ICC was also performed for each subject. All correlations were significant (P ≤ 0.01). The ICC of subjects ranged from 0.65 to 0.98 for M3 and from 0.63 to 0.89 for M1.

ICC results for activity categories are presented in Table 2. For all categories except "Antalgic therapy", association between estimate and measure of active time was significant (P ≤ 0.05) for M1 and M3. ICC varied from 0.68 to 0.95 for M3 and from 0.55 to 0.93 for M1. Ambulatory activities, like "Gait", "Stairs" and "Walking, outdoor", displayed the highest associations for M3, but not for M1.

**Table 2:** Intraclass correlation coefficients between estimates of active time and measure of active time for activity categories.

Activities	N	M3	M1
Gait	81	0.95 (0.93–0.97)	0.82 (0.74–0.88)
Balance, standing	50	0.76 (0.61–0.86)	0.82 (0.70–0.89)
Reinforcement	40	0.81 (0.66–0.89)	0.61 (0.37–0.77)
Weight bearing	39	0.83 (0.69–0.91)	0.62 (0.39–0.78)
Stairs	32	0.95 (0.90–0.98)	0.68 (0.44–0.83)
Prosthesis	25	0.92 (0.83–0.96)	0.85 (0.69–0.93)
Antalgic therapy	9	0.32* (-0.39–0.79)	0.29* (-0.42–0.78)
Walking, outdoor	6	0.92 (0.54–0.99)	0.93 (0.60–0.99)
Others	13	0.68 (0.23–0.89)	0.55 (0.03–0.84)

M3 represents the WBAN using three sensors and M1 represents the WBAN with only one sensor on the hip. Range of values presented in parentheses is 95% Confidence interval of the correlation. All correlations are statistically significant (P ≤ 0.05), except when marked with an asterisk (*). Values are presented as mean ± SD.

**Figure 5:**
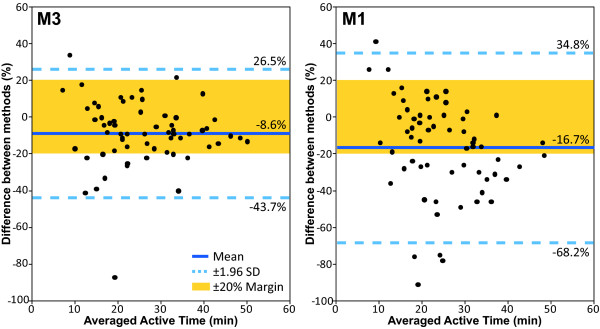
Bland-Altman plots of measure and estimate of active time for observed sessions. M3 and M1 are compared to time and motion (TM) analysis. On the Y-axis, differences between methods are expressed as: [(M-TM)/((TM+M)/2)*100]. On the X-axis, averaged active time is calculated as: [(M+TM)/2].

Differences between reference measure (TM) and estimates of active time (M1 and M3) are presented with Bland-Altman plots in Figure 5. Mean difference between methods are -8.6% ± 17.9% for M3 and -16.7% ± 26.3 forM1. Of the 62 paired values analyzed, 2 (3.2%) exceeded the Bland-Altman limits of agreement (95% CI = -43.7% to 26.5%) for M3, and 5 (8.1%) exceeded the Bland-Altman limits of agreement (95% CI = -68.2% to 34.8%) for M1. For M3, 80.6% (N = 50) of sessions were within the critical margins of agreement of ± 20%, with a range for subjects of 75% to 100%. For M1, this proportion was of 54.8% (N = 34) of sessions, with a range of 25% to 80% for subjects. Agreement levels with TM measures between M1 and M3 were significantly different for combined sessions (P ≤ 0.001) and for each subject (P ≤ 0.02), except for subject 1 (P ≤ 0.137).

Similar information is presented for activity categories in Table 3. For M3, activities that had the highest proportion of occurrences inside the critical margins of agreement of 20% were "Gait" (68%), "Stairs" (53%), "Prosthesis" (52%) and "Walking, outdoor" (50%). For M1, they were "Walking, outdoor" (67%), "Gait" (52%), "Prosthesis" (52%) and "Weight bearing" (43.6%). Differences with TM between M1 and M3 were significantly different (P ≤ 0.028) for "Gait", "Reinforcement", "Weight bearing" and "Stairs". For those mentioned above, the mean difference between WBANs was lower for M3 in all the categories except for "Stairs".

**Table 3:** Agreement and difference between estimates and measure of active time.

	**Inside 20% Critical Margin of Agreement (N)**			**Differences between methods (%)**		
**Activities**	Total	M3	M1	M3	M1	P-Value
Gait	81	55 (67.9%)	42 (51.9%)	-1.4 ± 32.7	-17.6 ± 50.7	<0.001
Balance	50	23 (46.0%)	19 (38.0%)	-18.9 ± 69.1	-18.9 ± 64.9	≤0.993
Reinforcement	40	18 (45.0%)	8 (20.0%)	-7.2 ± 83.1	-42.9 ± 101.6	<0.001
Weight bear.	39	19 (48.7%)	17 (43.6%)	-7.9 ± 65.2	-30.0 ± 83.5	≤0.003
Stairs	32	17 (53.1%)	9 (28.1%)	30.6 ± 59.3	18.8 ± 71.1	≤0.028
Prosthesis	25	13 (52.0%)	13 (52.0%)	23.5 ± 57.7	22.2 ± 62.0	≤0.633
Antalgic therapy	9	0 (0.0%)	0 (0.0%)	91.1 ± 104.2	94.1 ± 105.9	≤0.052
Walking, out.	6	3 (50.0%)	4 (66.7%)	33.0 ± 83.8	26.15 ± 91.8	≤0.511
Others	13	2 (15.4%)	1 (7.7%)	10.5 ± 94.5	16.5 ± 92.2	≤0.101

On the left side of the table, data reports the number of activities that were inside the ± 20% Critical Margin of agreement in the Bland-Altman Plots. On the right side, difference between measure (TM) and estimate (M) of active time are presented according to this formula: [(M-TM)/((TM+M)/2)*100]. Paired Sample T Test were used to evaluate the differences of agreement of both M3 and M1 with TM. Values are presented as mean ± SD.

## Discussion

The primary objective of this study was to explore the feasibility and accuracy of a WBAN composed of three accelerometer modules to estimate active time in physical therapy sessions. Our results show that WBAN estimates of active time using inputs from three accelerometer modules are 1) different on average by -8.7% ± 2.0% from TM measures of active time recorded throughout the length of stay and 2) highly correlated (ICC = 0.93, P < 0.001). Using only one accelerometer module instead of three leads to a lower correlation (ICC = 0.78, P < 0.001) and larger difference with TM (-16.4% ± 10.4%).

Time and motion measurements in the 62 sessions showed an average density (active time on total time) of 56.8% (52.6% for M3 estimates). Interestingly, our results revealed that active time and density varied considerably from one patient to another. Sessions density for patients ranged from 34.1% to 75.5%. In addition, the standard deviation was considerable for each patient (range: 8.7%–14.4%), which supports the hypothesis that total time of therapy is not an accurate portrait of active time, giving the fact that active time is not constant neither at the inter- nor intra-individual level.

A mean difference under 10% of TM measures gives strong support for the use of accelerometer-based WBANs to estimate active time in therapy. According to the literature, we chose a critical margin of agreement of 20% in order to consider that WBANs estimates were acceptable [16,31,32]. This margin is very conservative when considering the difficulties and logistics of obtaining data with work sampling and TM. For example, an error of at least 20% was reported when comparing measures form TM or work sampling [16]. Since TM is the most precise observation technique, a mean difference of less than 10% is therefore excellent. Moreover, these results put M1 estimates in another perspective. While less precise than M3, differences between M1 and TM are still acceptable. Therefore, if a WBAN system using three modules constitutes a burden under certain conditions, one module may be a viable alternative. Nevertheless, it should be noted that the range of differences for M1 is higher and that a study with more participants will be needed to validate its use with a wider range of patients.

Accelerometers seem to give better estimation of active time during ambulatory activities. In fact, gait, stairs and walking outdoor all have an ICC above 0.95 (P < 0.001). Concurrently, gait appears to have the lowest difference of agreement between accelerometers and TM. Interestingly, Horn et al. [15] found that spending more time in ambulatory activities lead to greater functional recovery and to a shorter length of stay. This reinforces the use of accelerometers as an interesting way to estimate physical activity. That being said, our results indicate that accelerometers are more precise on larger time frames to estimate active time: estimates for the full length of stay are more precise than for a single session, which estimates are in turn more precise than estimates for individual activities. Similar findings have been reported in the literature on physical activity in the population where validity of accelerometers increase with a higher number of observed days [20].

This study possesses several limitations. Having only five participants does not allow us to generalize our results to a larger population. In addition, we don't have inferential power and a sufficient sample size to evaluate the associations between active time and functional recovery. Furthermore, by only measuring active time in physiotherapy, observations cannot be expanded to other therapeutic approaches, like occupational therapy. Nevertheless, to our knowledge, this is the first study that tried to use accelerometers in the context of rehabilitation to estimate active time.

The fact that active time has yet to be established as an important determinant of functional recovery could be regarded as a limitation for this study. It is obvious that large-scale longitudinal designs are needed to study the theoretical association between physical activity (active time) and functional gains of patients. To this day, only short observational studies have been used to describe the activity profile of individuals in post acute rehabilitation centers. This illustrates the difficulty of making observations during longer periods of time, which is time and resources-consuming.

If the impact of physical mobilization on functional recovery is to be investigated, active time has to be evaluated during the entire day – not only during therapy sessions. As a matter of fact, therapies represent only a small fraction of total time in rehabilitation [4]. Evidences accumulate that rehabilitation programs alone are insufficient to provide enough active time for optimal functional recovery. Recent studies have suggested that physical activity done outside of supervised therapy may be more important, in term of time of mobilization, than therapies themselves [10,11,13]. Continuous observation of patients for long periods of time to assess the contribution of activities performed outside of traditional organized therapy would be impractical. On the other hand, accelerometers are small – about the size of a pager – and unobtrusive. They also have low power consumption; each module used in this study had autonomy of about 16 hours, which would make them very convenient to do ambulatory monitoring throughout the entire day. They could even be used as motivational devices by therapists, who could set goals of physical mobilization for their patients, outside of therapy.

## Conclusion

This study is the first step in a process to validate and use accelerometer-based WBAN to estimate active time in rehabilitation. Errors of estimate of active time using accelerometers are considerably inferior to most observation methods. While the use of three accelerometer modules appears to give more precise estimates of active time, the use of only one accelerometer module on the hip could still be an interesting alternative to observation methods and should be further investigated. Longitudinal studies in broader populations are now needed to verify the association between active time and outcomes of rehabilitation.

## Competing interests

The authors declare that they have no competing interests.

## Authors' contributions

SC and PB developed study concept and design. SC, PB and MH all participated in data analyses and interpretation. SC assumed manuscript preparation and the co-authors participated in revisions.

## Consent

Written informed consent was obtained from the patients for publication of this case report and any accompanying images. A copy of the written consent is available for review by the Editor-in-Chief of this journal.
